# Multi‐compartment *V/Q* lung modeling: Log normal distributions of inspired or expired alveolar gas?

**DOI:** 10.14814/phy2.16175

**Published:** 2024-09-01

**Authors:** Peter H. Scott, Thomas J. Morgan

**Affiliations:** ^1^ Intensive Care Department Mater Health Services Brisbane Queensland Australia; ^2^ University of Queensland Brisbane Queensland Australia; ^3^ Mater Research and University of Queensland Brisbane Queensland Australia

**Keywords:** alveolar gas, expired, inspired, log normal, MIGET, *V/Q* model

## Abstract

Using a 50‐compartment Python‐coded mathematical lung model, we compared mixed venous blood flow (*Q*) distributions and arterial oxygen tension/inspired oxygen fraction (PaO_2_/FiO_2_) relationships in lungs modeled with log normal distributions (LND) of inspired (*V*_I_) versus expired (*V*_A_) alveolar gas volumes. In lungs with normal *V/Q* heterogeneity, *Q* versus *V*_A_/*Q* and *Q* versus *V*_I_/*Q* distributions were similar with either approach, and PaO_2_/FiO_2_ sequences remained indistinguishable. In *V/Q* heterogeneous lungs at high FiO_2_, *V*_I_LND generated low *Q* versus *V*_A_/*Q* shoulders and some negative *V*_A_ units, while *V*_A_LND preserved *Q* versus *V*_A_/*Q* log normality by blood flow diversion from low *V*_I_/*Q* units. We managed *V*_I_LND‐induced negative *V*_A_ units either by shunt conversion (*V*_I_ decreased to 0) or *V*_I_ redistribution simulating collateral ventilation (*V*_I_ increased till *V*_A_ = 0). Comparing oxygen transfer: *V*_A_LND > *V*_I_LND (redistribution) > *V*_I_LND (shunt). In *V/Q* heterogeneous lungs *V*_A_LND and *V*_I_LND (redistribution) regained near optimal oxygen transfer on 100% oxygen, while impairment persisted with *V*_I_LND (shunt). Unlike *V*_A_LND, *V*_I_LND (redistribution) produced *Q* versus *V*_A_/*Q* distributions in *V/Q* heterogeneity compatible with multiple inert gas (MIGET) reports. *V*_I_LND (redistribution) is a physiologically–based MIGET–compatible alternative to West's original *V*_A_LND lung modeling approach.

## 1 INTRODUCTION

John West's landmark model of pulmonary gas exchange (West, 1969) equilibrates alveolar gas and mixed venous blood in a series of virtual lung units. The model architecture, described by West as “traditional,” is structured around log normal volume distributions of expired alveolar gas (*V*_A_) (West & Wagner, 1977).

Assigning log normal volume distributions to expired rather than inspired alveolar gas (*V*_I_) is an important detail. West cited theoretical and experimental data to support this approach (Farhi & Rahn, 1955; Lenfant & Okubo, 1968; Rahn, 1949), commenting to the effect that imposing log normal *V*_A_ distributions up front prevents negative *V*_A_ values, which he characterized as “physiologically meaningless” (West & Wagner, 1977).

Although effective, this strategy introduces a cause/effect conflict. The *V*_A_ output from each lung unit is a dependent variable subject to the input volumes of *V*_I_ and mixed venous blood (*Q*) and their subsequent equilibration. Mandating a unimodal log normal *V*_A_ distribution (*V*_A_LND) across lung units is an imposed independent outcome requiring specific adjustments of these proximal “feed in” factors.

The multiple inert gas technique (MIGET) is an investigative tool derived from the *V*_A_/*Q* model (Wagner, 2008; Wagner et al., 1974; 1977; 1978). MIGET readouts often report *V*_A_/*Q* distributions which deviate from unimodal log normality. Examples include *Q* versus *V*_A_/*Q* “shoulders” in low *V*_A_/*Q* lung regions, and bimodal distributions which typify MIGET reports in conditions such as the acute respiratory distress syndrome and asthma (Dantzker et al., 1979; Wagner et al., 1974, 1978; West, 1977).

When our group adapted West's *V/Q* lung model for a machine learning project (Morgan, Langley, et al., 2023; Morgan, Scott, et al., 2023), we chose log normal distributions of inspired gas (*V*_I_LND) as a more intuitive modeling format, knowing that with this approach *V*_A_ distributions can deviate from log normal, and that in some instances there will be negative *V*_A_ outputs from low *V*_I_/*Q* units (Morgan, Scott, et al., 2023). For simulation purposes, we redefined units with negative *V*_A_ outputs as having undergone atelectasis due to denitrogenation and remodeled them as shunt compartments (*V*_I_/*Q* = 0).

*V*_I_ redistribution was an alternative approach. Rather than reducing *V*_I_/*Q* ratios to zero to mimic alveolar collapse, *V*_I_ redistribution can be modeled by diverting small volumes of inspired gas from the total pool to increase the *V*_I_ of affected units, boosting negative *V*_A_ values to zero or higher (*V*_A_/*Q* ≥ 0). Redistributions along these lines simulate collateral ventilation, a phenomenon thought to occur via the Pores of Kohn (Desplechain et al., 1983) and facilitated by mechanical support from surrounding ventilated alveoli, a mechanism known as “alveolar interdependence” (Daly et al., 1975).

Accordingly, we tested the hypothesis that a *V*_I_LND approach incorporating the *V*_I_ redistribution option has physiological fidelity when set against the original West *V*_A_LND approach. The *V*_I_LND “shunt conversion” option was also included in the comparison.

## 2 METHODS

We devised two scenarios for hypothesis testing. In the first, focusing on pulmonary blood flow distribution (*Q* vs *V/Q*; log scale), we ascertained the effects at low and high FiO_2_ of the unmodified *V*_I_LND approach on post‐equilibration *Q* versus *V*_A_/*Q* relationships, comparing these with the corresponding retroactive influence of the West *V*_A_LND approach on pre‐equilibration *Q* versus *V*_I_/*Q* relationships. In the second scenario, we compared the oxygen transfer outcomes of both policies, dealing with negative *V*_A_ units induced by *V*_I_LND by trialing the two supplementary options:

1. In Option 1, all negative *V*_A_ units were subjected to shunt conversion by reducing *V*_I_ to zero (*V*_I_/*Q* = 0) to mimic across the board denitrogenation‐induced collapse.
2. In Option 2, the *V*_I_ inputs of affected units were boosted from the inspired gas pool until *V*_A_/*Q* = 0, to model a limited *V*_I_ redistribution.

A 50‐compartment Python program was developed capable of switching between West's *V*_A_LND approach and the *V*_I_LND approach without changing inputs (see Appendix S1).

Fixed model inputs were:

Blood hemoglobin concentration (Hb) 15 g/dL.

Arterial PCO_2_ 40 mm Hg.

Base excess 0 m*E*q/L (Siggaard‐Andersen, 1977).

CO_2_ production (*V*CO_2_) 200 mL/min.

O_2_ consumption (*V*O_2_) 250 mL/min.

Standard P50 26.8 mm Hg (Morgan, 1999).

Cardiac output 5 L/min.

Shunt 0 L/min.

FiO_2_ 0.3 or 0.9 (Scenario 1 only).

### 2.1 Scenario 1

Graphical representations (log scale) were created of *Q* versus the relevant *V/Q* ratios of the two policies at FiO_2_ = 0.3 and 0.9 for:

Log standard deviation (Log SD) = 0.4.

Log SD = 1.1.

Log SD = 1.8.

### 2.2 Scenario 2

Arterial PO_2_ (PaO_2_) values and venous admixture calculations were computed from FiO_2_ = 0.2 to FiO_2_ = 1.0 at FiO_2_ intervals of 0.05 for the above three log SD values via:

1. The West *V*_A_LND approach
2. The *V*_I_LND approach modified so that:

- Units with negative *V*_A_ values were converted to shunt (*V*_I_/*Q* = 0) (Option 1).
- *V*_I_/*Q* ratios of negative *V*_A_ units were increased by redistributing inspired gas so that *V*_A_/*Q* = 0 (Option 2).

## 3 RESULTS

### 3.1 Scenario 1

**FIGURE 1:**
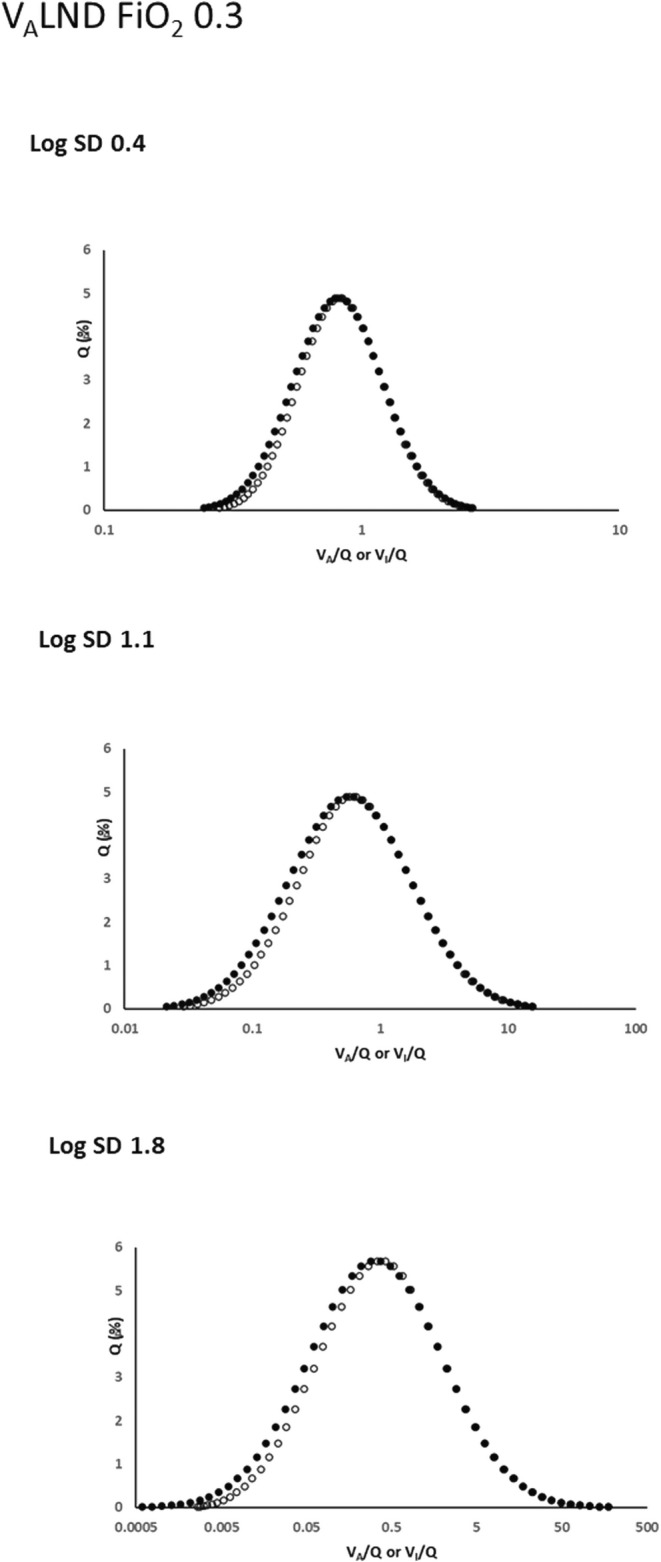
Effects of West's *V*_A_LND approach on pre‐equilibration *Q* versus *V*_I_/*Q* relationships at a low inspired oxygen concentration (FiO_2_ = 0.3). Closed circles: *Q* versus *V*_A_/*Q* maintained as a log normal distribution. Open circles: *Q* versus *V*_I_/*Q*: Remains close to log normal as log SD increases, but with some unilateral rightward shift on the left side of the log normal mean.

Figure 1 demonstrates that at FiO_2_ = 0.3 the West *V*_A_LND approach produces almost identical *Q* versus *V*_A_/*Q* and *Q* versus *V*_I_/*Q* relationships at log SD = 0.4, with slight reduction in concordance as log SD increases.

**FIGURE 2:**
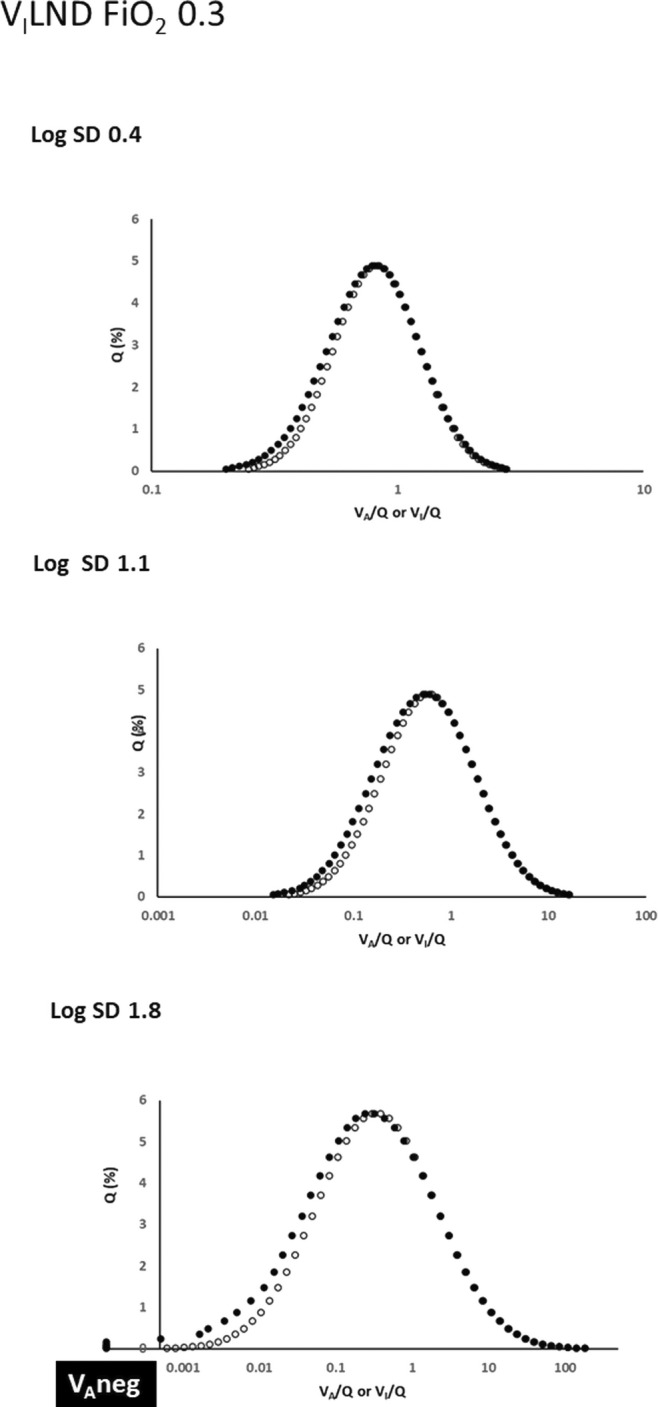
Effects of the *V*_I_LND approach on post‐equilibration *Q* versus *V*_A_/*Q* relationships at FiO_2_ = 0.3. Open and closed circles as in Figure 1, but with *Q* versus *V*_I_/*Q* (open circles) maintained as log normal. There is an elevated *Q* versus *V*_A_/*Q* ‘tail’ on the left of the log normal mean as log SD increases, indicating increased blood flow to low *V*_A_/*Q* units. At log SD = 1.8 there is also blood flow to 7 negative *V*_A_ units.

Similarly, as shown in Figure 2, the *V*_I_LND approach at FiO_2_ = 0.3 produces almost identical *Q* versus *V*_A_/*Q* and *Q* versus *V*_I_/*Q* distributions at log SD = 0.4 and 1.1. At log SD = 1.8 blood flow to low *V*_A_/*Q* units has more clearly increased, and now includes flow to 7 negative *V*_A_ units of the 50‐compartment model.

**FIGURE 3:**
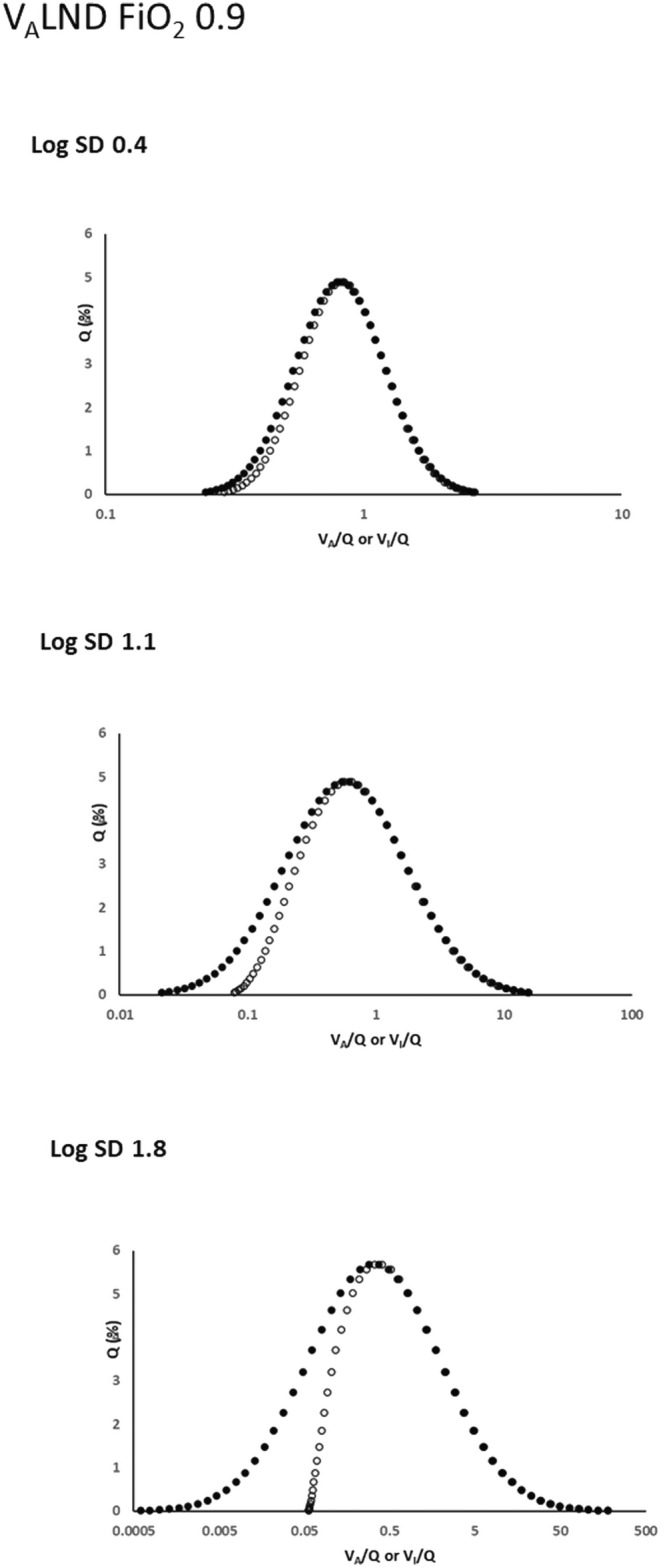
Effects of West's *V*_A_LND approach on *Q* versus *V*_I_/*Q* pre‐equilibration relationships at a high inspired oxygen concentration (FiO_2_ = 0.9). Open and closed circles as in Figure 1 with *Q* versus *V*_A_/*Q* (closed circles) maintained as log normal. As log SD increases there is now significant and progressive unilateral deviation from a log normal *Q* versus *V*_I_/*Q* relationship by rightward shifts on the left of the log normal mean.

Figure 3 shows that the West *V*_A_LND approach operating at FiO_2_ = 0.9 causes a unilateral rightward shift in pre‐equilibration blood flow away from low *V*_I_/*Q* values as log SD increases.

**FIGURE 4:**
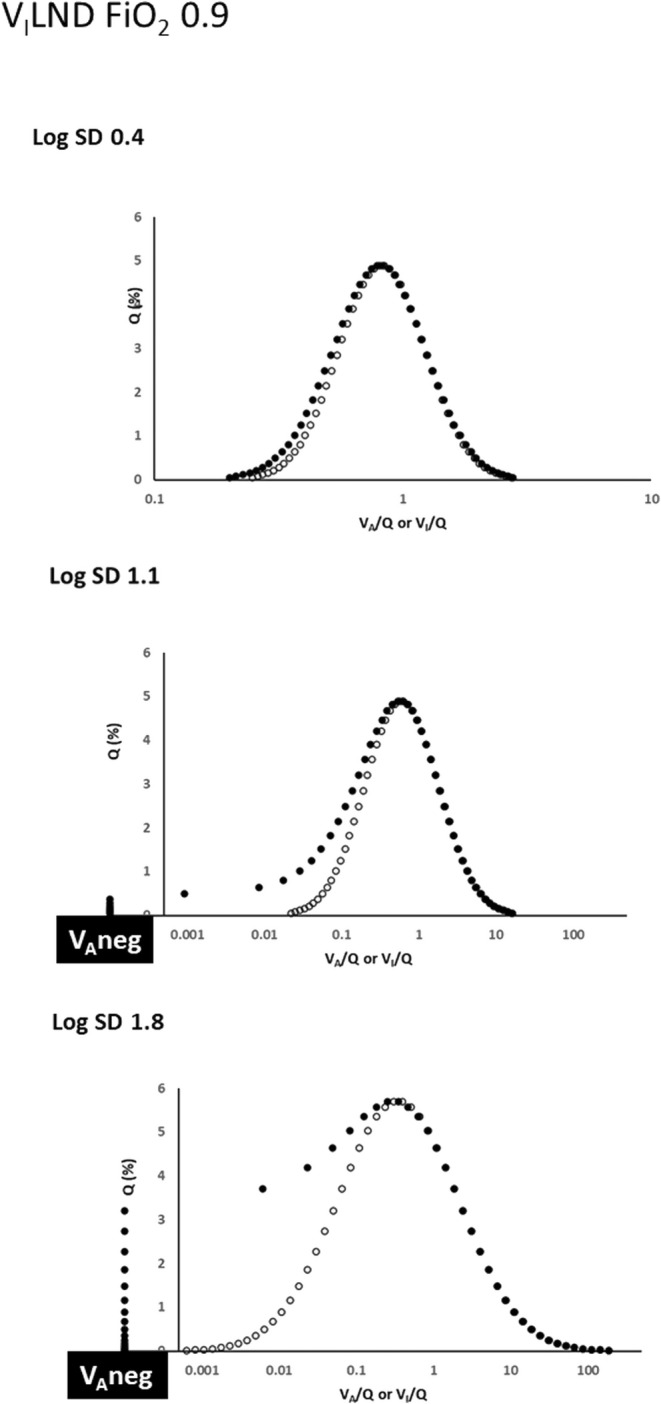
Effects of the *V*_I_LND approach on *Q* versus *V*_A_/*Q* post‐equilibration relationships at high FiO_2_ (0.9). Open and closed circles as in Figure 1, with *Q* versus *V*_I_/*Q* (open circles) maintained as log normal. There is a progressive *Q* versus *V*_A_/*Q* “shoulder” on the left of the log normal mean as log SD increases, indicating escalating blood flow to low *V*_A_/*Q* units. There are now major flows to negative *V*_A_ units.

Figure 4 shows that with the *V*_I_LND approach operating at FiO_2_ = 0.9, there is unilateral upward displacement of *Q* versus *V*_A_/*Q* relationships (on the low *V/Q* side) as log SD increases, indicating a diversion of blood flow to low *V*_A_/*Q* units. At log SD = 1.1, blood is also flowing to 7 negative *V*_A_ units, amounting to 1.3% of cardiac output. At log SD = 1.8, 18 of the 50 compartments have become negative *V*_A_ units, receiving nearly 16% of cardiac output in total.

### 3.2 Scenario 2

**FIGURE 5:**
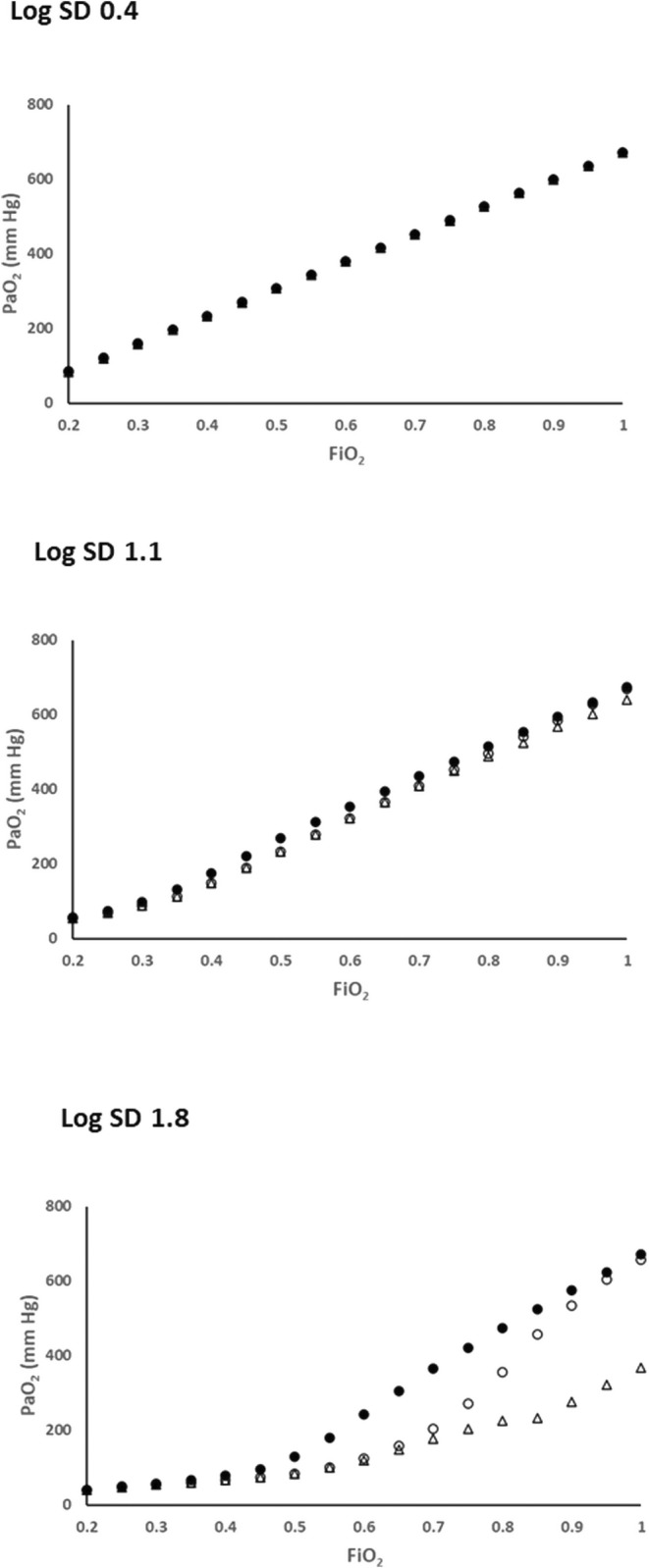
Relationships between FiO_2_ and PaO_2_ for log SD = 0.4, 1.1 and 1.8. Closed circles: West's *V*_A_LND approach. Open triangles: *V*_I_LND approach with conversion of negative *V*_A_ units to shunt (*V*_I_/*Q* = 0) (Option 1). Open circles: *V*_I_LND approach with redistribution of inspired gas to negative *V*_A_ units so that *V*_A_/*Q* = 0 (Option 2). PaO_2_ values vs FiO_2_ are close to identical at log SD = 0.4 but diverge for all policies at higher log SD values, with West's *V*_A_LND approach achieving the highest oxygen transfer at each FiO_2_.

**FIGURE 6:**
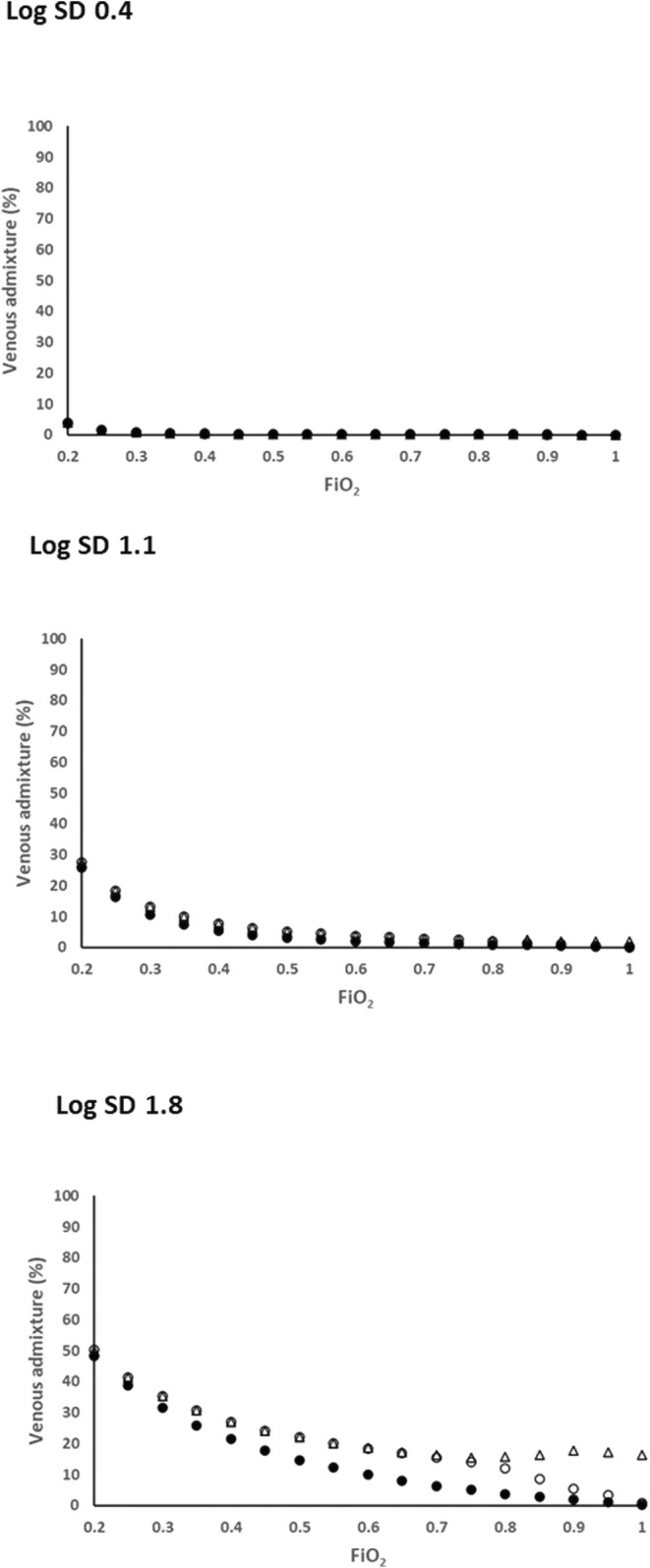
Relationships between FiO_2_ and venous admixture for log SD = 0.4, 1.1 and 1.8. Closed circles: West's *V*_A_LND policy; Open triangles: *V*_I_LND policy with conversion of negative *V*_A_ units to shunt (*V*_I_/*Q* = 0) (Option 1); Open circles: *V*_I_LND policy with redistribution of inspired gas to negative *V*_A_ units so that *V*_A_/*Q* = 0 (Option 2). Venous admixture values are close to identical for all policies at log SD = 0.4 and log SD = 1.1, and diverging at log SD = 1.8. For log SD = 1.8 and FiO_2_ = 1.0, venous admixture persists at approximately 20% for Option 1, but is verging on zero for *V*_I_LND plus Option 2 and for West's *V*_A_LND policy.

Figure 5 shows that at log SD = 0.4 the three strategies: *V*_A_LND, *V*_I_LND with Option 1 (shunt conversion of negative *V*_A_ units), and *V*_I_LND with Option 2 (limited *V*_I_ redistribution to negative *V*_A_ units) display superimposed PaO_2_ versus FiO_2_ relationships throughout the FiO_2_ range. At log SD = 1.1, concordance is reduced, and is lost at log SD =1.8, with West's *V*_A_LND approach producing consistently higher PaO_2_ values across the FiO_2_ range. At log SD = 1.8 and higher FiO_2_ settings, PaO_2_ values achieved by the *V*_I_LND with the Option 2 redistribution strategy recover toward the West PaO_2_/FiO_2_ curve, reaching near parity at FiO_2_ = 1.0. As a result, both achieve venous admixture values close to zero at this FiO_2_ (Figure 6). By contrast shunt conversion (Option 1) causes persistently low PaO_2_ values which diverge progressively from those of the other two methods as FiO_2_ increases, while corresponding venous admixture calculations remain significant at around 20%.

## 4 DISCUSSION

We explored two scenarios in which the *V*_I_LND lung modeling approach, versions of which were used previously to simulate bedside diagnostic data for machine learning (Morgan, Langley, et al., 2023; Morgan, Scott, et al., 2023), was evaluated in parallel with West's original *V*_A_LND approach (West & Wagner, 1977). For PaO_2_ calculations, negative *V*_A_ units encountered with *V*_I_LND underwent *V*_I_ redistribution (*V*_I_ increased till *V*_A_ = 0) or shunt conversion (*V*_I_ decreased to 0).

In contrast to the West approach which fixes expired alveolar gas distributions “post‐equilibration” as log normal, the *V*_I_LND adaptation is applied “pre‐equilibration” to fix inspired alveolar gas distributions as log normal. The Python program developed for the comparison can alternate between the two policies, so that a log normal distribution of alveolar gas can be switched to either pole of the *V*_I_/*V*_A_ axis. The distribution at the non‐dominant pole becomes the dependent variable.

In this evaluation, several aspects impacting the physiological fidelity of both approaches are evident. With either approach operative at log SD = 0.4 (representing the low *V/Q* heterogeneity of healthy lungs), *Q* versus *V*_I_/*Q* and *Q* versus *V*_A_/*Q* distributions remain undistorted close copies at both low and high FiO_2_ (Figures 1, 2, 3, 4). No negative *V*_A_ units are produced with *V*_I_LND. Consequently, as shown in Figure 5, *V*_A_LND/*V*_I_LND switches at this healthy *V/Q* distribution make little difference to gas exchange across the FiO_2_ range. It is thus reasonable to assume that inspired and expired alveolar gas display similar distributions when modeling healthy lungs whether using *V*_A_LND or *V*_I_LND. Given previous findings (Farhi & Rahn, 1955; Lenfant & Okubo, 1968), these are likely to be log normal or close approximations.

However, Figures 1, 2, 3, 4, 5 show that *V*_A_LND/*V*_I_LND interchangeability is lost at higher log SD values characteristic of conditions with increased *V/Q* heterogeneity such as chronic obstructive pulmonary disease. With either approach operative, loss of concordance between *Q* versus *V*_I_/*Q* and *Q* versus *V*_A_/*Q* distributions is most evident in the current data at FiO_2_ = 0.9 (Figures 3 and 4), along with a separation of *V*_A_LND and *V*_I_LND–generated PaO_2_/FiO_2_ sequences (Figure 5).

As outlined in the Introduction, West's *V*_A_LND approach eliminates negative *V*_A_ units by the direct imposition of unimodal log normal expired gas distributions across lung units. How this modifies the “feed in” pre‐equilibration factors can be seen in Figures 1 and 3, where *Q* distributions are selectively shifted from lower *V*_I_/*Q* units, most prominently at high log SD scenarios at the higher FiO_2_ setting of 0.9. Put simply, West's *V*_A_LND approach has the effect of directing mixed venous blood away from poorly ventilated alveoli, whereas under similar conditions the *V*_I_LND approach does the opposite by directing blood flow towards low (and negative) *V*_A_/*Q* units.

In some respects, West's *V*_A_LND approach resembles the feedback action of hypoxic pulmonary vasoconstriction (HPV), since HPV operates on the same broad principle of restricting mixed venous blood flow to poorly ventilated units (Dantzker et al., 1974; Grant et al., 1976). One difference is that HPV alters pulmonary blood flow distribution via a vasoconstrictive loop sensitive to a composite alveolar/mixed venous PO_2_ value (Marshall & Marshall, 1983). Another is that whereas higher FiO_2_ settings reduce rather than magnify HPV‐induced pulmonary blood flow redistribution (Marshall & Marshall, 1983), the opposite occurs with the *V*_A_LND approach (Figure 3). In short, West's *V*_A_LND approach is not a model of HPV.

As mentioned, the effect of *V*_I_LND on blood flow distribution (Figures 2 and 4) is such that at higher log SD values flow increases through low *V*_A_/*Q* units in configurations similar to MIGET “shoulders” reported in the lungs of older subjects (Wagner et al., 1974). At the same time some mixed venous blood perfuses negative *V*_A_ units, more so at high FiO_2_. Although *V*_A_LND abolishes “physiologically meaningless” negative *V*_A_ values (West & Wagner, 1977), it does so by forcing idiosyncratic reconfigurations of incoming *V*_I_ distributions (Figure 3), in effect transferring biological implausibility “upstream.” An advantage of the *V*_I_LND approach is that negative *V*_A_ values when encountered can be reconfigured directly by responses more consistent with known physiology.

Two examples were evaluated in the present study. Option 1 (converting negative *V*_A_ units into shunt) causes a severe gas exchange deficit (Figure 5) and persistently high venous admixture (Figure 6) all the way to 100% oxygen. In contrast, with Option 2 (increasing *V*_A_/*Q* to zero by *V*_I_ redistribution) an initial deficit in oxygen transfer relative to the West approach is followed by a steady recovery with increasing FiO_2_ so that both achieve near zero venous admixture at FiO_2_ = 1.0 (Figure 6).

**FIGURE 7:**
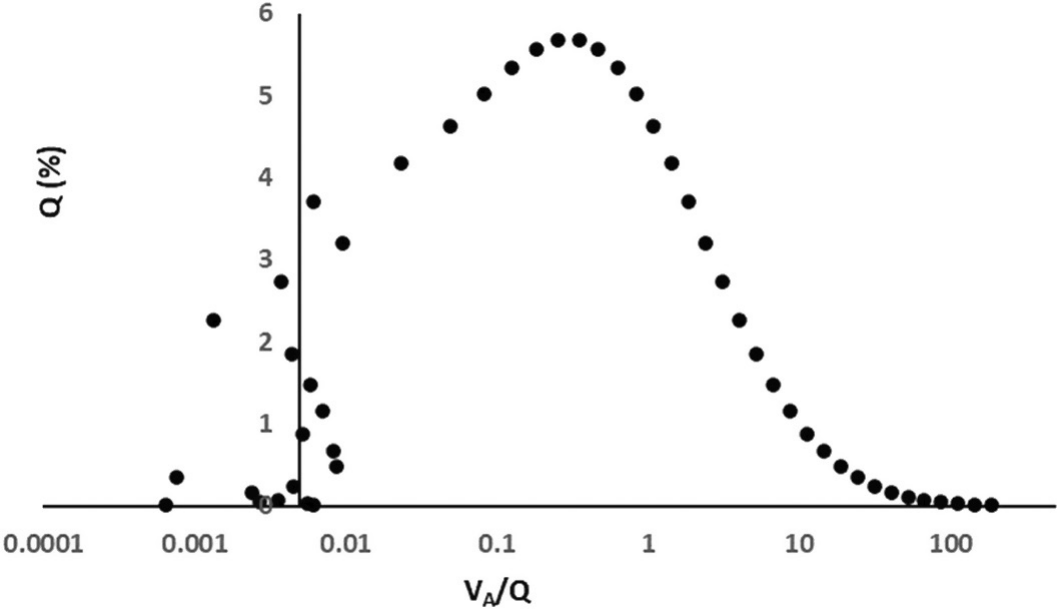
Illustration of extensive *V*_I_ redistribution. Log SD = 1.8. FiO_2_ = 0.9. Prior to redistribution 20% of mixed venous flow was to negative *V*_A_ units. *V*_I_ redistribution to these units was randomized to achieve *V*_A_/*Q* values between zero and 0.01. The result is 8% mixed venous blood flow to units with *V*_A_/*Q* < 0.005 (all data points to the left of the Y axis), interpretable by MIGET as shunt flow. The rest (all data points to the right of the Y axis) would potentially be processed by the MIGET smoothing algorithm as a bimodal distribution.

Of note, MIGET analysis using the standard inert gas mix, which includes sulfur hexafluoride as the least soluble gas, will report blood flows to units with *V*_A_/*Q* < 0.005 as shunt (*V*_I_/*Q* = 0) (Wagner et al., 1974). Hence negative *V*_A_ units redistributed to *V*_A_/*Q* = 0 as in Option 2 fall into the MIGET “shunt” category, whereas more extensive *V*_I_ redistribution generating *V*_A_/*Q* values ≥0.005 (Figure 7) and subjected to MIGET's smoothing algorithm (Wagner, 2008) would likely be reported as bimodal or even trimodal *Q* versus *V*_A_/*Q* distribution patterns. This raises the possibility that multi‐modal MIGET reports are flagging extensive alveolar gas redistribution.

Our group originally adopted Option 1 for Machine Learning data simulations (Morgan, Langley, et al., 2023; Morgan, Scott, et al., 2023). However converting negative *V*_A_ units into shunt across the board fails to replicate reported improvements in oxygen transfer as FiO_2_ increases in *V/Q* heterogeneous lungs (Wagner et al., 1977). These are more closely reproduced with Option 2 (*V*_I_ redistribution).

Conversely, while marked facilitation of oxygen transfer is known to occur when *V/Q* heterogeneous lungs receive 100% oxygen (Wagner et al., 1977), improvements fall short of the virtual elimination of impairment shown in Figures 5 and 6, where both *V*_I_LND plus *V*_I_ redistribution and West's *V*_A_LND approach predict PaO_2_ >670 mm Hg at FiO_2_ = 1.0 and close to zero venous admixtures. To our knowledge, even in individuals with healthy lungs 600 mm Hg is the highest documented PaO_2_ recorded at FiO_2_ = 1.0, achieved after 35 min equilibration with 100% oxygen and negligible end tidal N_2_ measurements (Wagner et al., 1974).

Suggested explanations for this apparent underperformance have included incomplete denitrogenation (Wagner et al., 1974), leaks in the delivery system, and negative measurement bias (Wagner et al., 1977). However, the shortfalls could also reflect the contribution of actual physiologic phenomena, for example, new onset atelectasis or “shunt—like” effects from high capillary blood velocity in some units (Domino et al., 1993; Miserocchi et al., 2022). Resultant oxygen transfer shortfalls can be accommodated in West's *V*_A_LND approach and the *V*_I_LND plus *V*_I_ redistribution approach by factoring in an additional shunt (*V*_I_/*Q* = 0) sufficient to match the venous admixture.

## 5 CONCLUSIONS

Based on data generated by a purpose‐designed 50 compartment *V/Q* lung modeling “switch” program we conclude the following:

- In lungs with a normal degree of *V/Q* heterogeneity, the *V*_I_LND approach and West's original *V*_A_LND model are essentially interchangeable, generating close to identical oxygen transfer outcomes and similar *Q* versus *V*_A_/*Q* and *Q* versus *V*_I_/*Q* relationships displaying approximately log normal configurations.
- In lungs with increased *V/Q* heterogeneity, there are important differences.
- The *V*_A_LND approach avoids negative *V*_A_ values by direct imposition of unimodal log normal *V*_A_ distributions, forcing idiosyncratic reconfigurations of incoming *Q* versus *V*_I_/*Q* distributions. By mandating post‐equilibration *V*_A_ distribution *V*_A_LND cannot accommodate MIGET deviations from *Q* versus *V*_A_/*Q* log normality or multi‐modal patterns.
- By contrast, the *V*_I_LND approach under *V/Q* heterogeneous conditions shifts blood flow towards lower *V*_A_/*Q* units and can cause “non‐physiological” blood flow through negative *V*_A_ units. These can be resolved by increasing inspired gas distribution to negative *V*_A_ units until *V*_A_ ≥0 simulating *V*_I_ redistribution by collateral ventilation. Such adjustments are consistent with known pathophysiological mechanisms, MIGET reports, and oxygen transfer profiles. Notably:
  - *V*_I_ redistribution to *V*_A_/*Q* < 0.005 produces *Q* versus *V*_A_/*Q* distributions which would be reported by MIGET as shunt.
  - *V*_I_ redistribution to *V*_A_/*Q* ≥ 0.005 produces *Q* versus *V*_A_/*Q* distributions potentially reported by MIGET in bimodal or even trimodal formats.
- Multimodal *Q* versus *V*_A_/*Q* MIGET reports may thus in some cases signify considerable redistribution of inspired alveolar gas rather than “true” multi‐modality. In such cases, redistribution at lower levels could have further increased MIGET shunt estimates above actual values.
- MIGET interpretations along these lines have as yet unclear implications for the deployment of management strategies such as high flow intranasal therapy, prone positioning, and lung recruitment maneuvers.
- A clinical study of bedside MIGET evaluations versus concurrent *V*_I_LND (redistribution) lung modeling conducted using Machine Learning methodology (Morgan, Scott, et al., 2023) would be instructive as a first step.

## AUTHOR CONTRIBUTIONS

Thomas J. Morgan devised the project and wrote the first draft of the main manuscript text. Peter H. Scott devised the Python *V/Q* “switch” program which generated the data and wrote the Supplementary Material text. Both revised and approved final versions.

## FUNDING INFORMATION

This project was supported by Departmental Funds.

## CONFLICT OF INTEREST STATEMENT

The authors declare that they have no competing interests.

## Supporting information


Data S1.

## Data Availability

Expressions of interest for data availability can be directed to the corresponding author. An online version of the lung model is under development.
