# Supplementary material for: Multi‐compartment V/Q lung modeling: Log normal distributions of inspired or expired alveolar gas?
Source: Physiol Rep. 2024 Sep 1;12(17):e16175. doi: 10.14814/phy2.16175 (PMC11366441; doi:10.14814/phy2.16175)
Supplement: Supplementary file 1 — Data S1. [file PHY2-12-e16175-s001.docx]

**Appendix**

**The Python coded mathematical lung model**

Python 3.8.3 was used to develop a mathematical model based upon the framework presented by West et al (West, 1977) and as described previously (Morgan et al., 2023).

The model is set up with multiple compartments that feature spreads of ventilation and perfusion according to log normal distributions. The setup may be achieved with one of two approaches requiring the following parameters respectively:

1. As described originally by West, parameters for total alveolar ventilation (V), total blood flow (Q), log standard deviation of the distribution of ventilation (log SD of V), log standard deviation of the distribution of blood flow (log SD of Q) and the mean ratio of ventilation to perfusion (mean VQ ratio).
2. An adapted approach requiring parameters for Q, mean VQ ratio and log standard deviation of the distribution of VQ ratios (log SD of VQ). In this approach, a normal distribution of blood flow to compartments is set up with a spread defined by the value of log SD of VQ with maximum flow to the compartment with the mean VQ ratio. Ventilation for each compartment is dependent upon the blood flow and VQ ratio assigned to the compartment. The log SD of VQ correlates with the difference between log SD of V and log SD of Q in the previous approach. In practice, West et al (West, 1977), alluding to the feasibility of this approach, would set log SD of one parameter to 0.1 and adjust the other accordingly.

Additional parameters required to define the model are oxygen consumption (VO_2_), CO_2_ production (VCO_2_), VO_2_:VCO_2_ ratio (R), fractional inspired oxygen (FiO_2_), barometric pressure, partial pressure of inspired CO_2_ (PiCO_2_), hemoglobin concentration (Hb), base excess (BE), partial pressure of O_2_ at 50% hemoglobin saturation under standard conditions (p50st), total blood flow, percentage of cardiac output of added shunt and the number of compartments. Calculations for the model occur at a temperature of 37^o^ C.

The model can be constrained to achieve a selected value for partial pressure of CO_2_ in arterial blood (PaCO_2_) or, alternatively, a value for arterial pH. The model adjusts to achieve nitrogen balance, such that no net uptake or elimination of nitrogen occurs. As inspired and expired volumes to a lung unit usually differ, the model can be set for the log normal distribution of V to be applied to compartmental inspired minute volumes (V_I_), or to expired minute volumes (V_A_). The corresponding calculated V_A_ or V_I_ values, respectively, would not fit a normal distribution exactly. As defined, the V_A_ model cannot have negative values for compartmental expired volumes, however, using the V_I_ approach this might occur with high FiO_2_. The V_I_ model can handle negative expired volumes by (a) accepting these values (b) collapsing the lung units converting to shunt, or (c) restoring the expired volume to zero by augmenting the inspired volume with fresh or expired gas.

Having set the model parameters, the following calculations are required:

1. Values for mixed venous partial pressure of O_2_ (mixed venous PO_2_) and mixed venous partial pressure of CO_2_ (mixed venous PCO_2_) are iteratively determined. An initial estimate is made for the mixed venous partial pressure of nitrogen (mixed venous PN_2_) based upon the inspired partial pressure of nitrogen. The iterative process minimizes the difference between summed values of uptake/excretion of O_2_/CO_2_ from all compartments and known values for VO_2_ and VCO_2_. This requires a further iterative subprocess:
   1. Postcapillary blood gas tensions and alveolar gas tensions for each lung unit are iteratively determined. The iteration minimizes the difference between the results of the following two calculations:
      1. Gas contents of post capillary blood according to postcapillary gas tensions.
      2. Gas contents of post capillary blood according to addition of mixed venous contents and uptake/excretion of O_2_ and CO_2_. The latter is determined from the inspired and alveolar gas concentrations and the VQ ratio [Equations 13-14 below]. Standard equations of blood content yield results in standard temperature and pressure dry (STPD) while measurements of respiratory gas volumes are given at body temperature and pressure saturated (BTPS), thus an adjustment is required prior to calculating the addition (West, 1977).
2. The mixed venous PN_2_ is iteratively adjusted, and Step 1 sequences rerun until nitrogen balance is zero.
3. The contents in post capillary blood flowing from lung units added to the contents in shunted blood will give the contents of arterial O_2_ and CO_2_ and corresponding arterial PO_2_ (PaO_2_) and PaCO_2_ values. Due to interdependence, an iterative approach is used.
4. To target a selected value for PaCO_2_ or pH, V is adjusted, and the adjustment spread across the lung units according to the normal distribution. The sequence from Step 1 is then repeated until the target PaCO_2_ or pH is achieved. The effect of this adjustment is to alter the mean VQ ratio. For the setup of compartments using preset V, the value for V is adjusted.
5. For each lung unit, if the V_I_ model is being used, the resultant expired volume is derived based upon the net of VO_2_ and VCO_2_ and vice versa for V_A_ models.
6. For the V_I_ models, if compartments have negative expired volumes, the following approaches can be selected:
   1. The negative volumes are accepted.
   2. The compartmental V_I_ is set to zero and blood flow to the compartment is considered a component of venous admixture. Recalculation follows from Step 1.
   3. V_I_ to the affected compartments is supplemented followed by recalculation from Step 1. This process is repeated until compartmental V_A_ values are zero. While the volumes redistributed are small, the consequences for oxygenation can be significant. Of note, whether redistribution occurs with fresh or expired gas makes little difference given this phenomenon occurs at high FiO_2_. At high FiO_2_, the O_2_ content of expired gas is also very high.

Blood CO_2_ content (CbCO_2_) is calculated by applying the Douglas equation (Douglas et al., 1988) (Equations 2 and 3) rather than via the Kelman sub-routine used by West (Kelman, 1967). The Douglas equation is a 1988 update of the ‘McHardy – Visser’ equation (McHardy, 1967), with new constants derived from experiments on volunteers. Agreement with these experimental data was closer than with the relevant Kelman sub-routine.

Hemoglobin – oxygen dissociation is modelled using Kelman`s procedure as used by West. An alternative approach using Siggaard-Andersen’s tan H equation (Siggaard-Andersen et al., 1993) gives similar results. (Equations 8 and 8a)

To determine blood pH, an iterative approach using the Van Slyke equation (Equation 6) (Siggaard-Andersen, 1977) is applied. pH is iteratively calculated knowing PCO_2_, Hb and BE. Having determined pH, it is straightforward to calculate CO_2_ content using the Douglas equation and to calculate O_2_ saturation as above (Siggaard-Andersen et al., 1993).

The multiple inert gas elimination technique (MIGET) approach usually has 50 gas exchanging compartments (Wagner, 2008). In developing a mathematical model, West found increasing model compartment numbers above 10 had minimal effect on PaO_2_ and PaCO_2_ (West, 1969). However, use of a computerized lung model readily enables larger numbers of compartments.

Core Equations

1. CbO_2_ (Blood oxygen content in ml/dL)

$$CbO_{2}=1.34.Hb\times S+0.003\times PO_{2}$$

Hb = Blood hemoglobin concentration (g/dL)

S = fractional saturation of hemoglobin with oxygen

PO_2_ = partial pressure of oxygen in blood (mm Hg)

1. CpCO_2_ (Plasma CO_2_ content in ml/dL)

$$CpCO_{2}=2.226\times0.03.PCO_{2}.\left( 1+{10}^{pH-6.1} \right)$$

PCO_2_ = partial pressure of carbon dioxide in blood (mm Hg)

1. CbCO_2_ (Blood CO_2_ content in ml/dL)

$$CbCO_{2}=CpCO_{2}\times\left[ 1-\frac{0.0289\times Hb}{\left( 3.352-0.456\times S \right).\left( 8.142-pH \right)} \right]$$

1. Bicarb (Plasma bicarbonate in mmol/L)

$$Bicarb=0.03\times PCO_{2}\times{10}^{pH-6.1}$$

2. BE (Base excess in mEql/L) (Siggaard-Andersen, 1977)

$$BE=\left( 1-0.0143\times Hb \right)\times\left[ Bicarb-24.4+\left( 1.4276\times Hb+7.7 \right)\times\left( pH-7.4 \right) \right]$$

3. PCO_2_ from pH and BE (van Slyke equation)

$$PCO_{2}=\frac{\left[ \frac{BE}{1-0.143\times Hb}-\left( 1.428\times Hb+7.7 \right)\times\left( pH-7.4 \right)+24.4 \right]}{0.03\times{10}^{pH-6.1}}$$

4. P50act (actual P50 is PO_2_ in mm Hg at 50% hemoglobin-oxygen saturation)

$$P50act=P50st\times{10}^{-0.48\times\left( pH-7.4 \right)}$$

P50st is standard P50 (the P50 at pH = 7.4 and PCO_2_ = 40 mm Hg).

1. Fractional saturation of hemoglobin (S) using Tanh (Siggaard-Andersen et al., 1993)

$$logitS=1.87+LN\left( \frac{PO_{2}}{1.955\times P50act} \right)+3.5\times TanH\left[ \frac{LN\frac{{PO}_{2}}{1.955\times P50act}}{3.5} \right]$$

$$S=\frac{EXP\left( logitS \right)}{1+EXP\left( logitS \right)}$$

LN is the natural logarithm.

TanH is the hyperbolic tangent function.

EXP (x) represents e raised to the power x.

8a. Kelman`s procedure for fractional saturation of haemoglobin (S)(Kelman, 1967)

A1 = -8532.229, A2=2121.401, A3= -67.07399, A4= 935960.9

A5= -31346.26, A6=2396.167, A7= -67.10441

B=0.43429(LN(40)-LN(PCO_2_))

C=PO_2_ x 10^(0.024(37-Temp)+0.4(pH-7.4)+.06 x B)^

D =C x 26.8/(26.8 + DP50)

if (D-10)<0:

S = 0.003683 x D + 0.000584 x D x D

else:

S = (D*(D*(D*(D+A3)+A2)+A1))/(D*(D*(D*(D+A7)+A6)+A5)+A4)

PCO_2_: partial pressure carbon dioxide

PO_2_: partial pressure oxygen

Temp: temperature

DP50: difference to normal p50 (mmHg)

1. VenAd (Venous admixture as %)

$$CcO_{2}=1.34.Hb\times SAO_{2}+0.003\times P{AO}_{2}$$

$$CvO_{2}=1.34.Hb\times SvO_{2}+0.003\times P{vO}_{2}$$

$$CaO_{2}=1.34.Hb\times SaO_{2}+0.003\times P{aO}_{2}$$

$$VenAd=100\times\frac{CcO_{2}-CaO_{2}}{CcO_{2}-CvO_{2}}$$

PAO_2_ = Partial pressure of oxygen in ideal alveoli (mm Hg)

CcO_2_ = Oxygen content of “ideal” pulmonary capillary after equilibration (ml/dL)

CvO_2_ = Oxygen content of mixed venous blood (ml/dL)

CaO_2_ = Oxygen content of arterial blood (ml/dL)

SAO_2_ = Fractional saturation of hemoglobin with oxygen in “ideal” pulmonary capillary blood after equilibration

SvO_2_ = Fractional saturation of hemoglobin with oxygen in mixed venous blood

SaO_2_ = Fractional saturation of hemoglobin with oxygen in arterial blood

1. PiO_2_ (Inspired partial pressure of O_2_ in mm Hg)

$$PiO_{2}=\left( BP-47 \right)\times FiO_{2}$$

2. PiN_2_ (Inspired partial pressure of N_2_ in mm Hg)

$$PiN_{2}=BP-47-PiO_{2}$$

3. CvN_2_ (Mixed venous N_2_ content - ml N_2_/dL)

$$CvN_{2}=0.0017\times PvN_{2}$$

1. By conservation of mass:

Vi x FIO_2_ – Va x FAO_2_ = Q (CaO_2_ - CvO_2_)

Vi x FICO_2_ – Va x FACO_2_ = Q (CaCO_2_ – CvCO_2_)

Vi x FIN_2_ – Va x FAN_2_= Q (CaN_2_ – CvN_2_)

1. Components of dry gas in alveolar and inspired gas:

FAO_2_ + FACO_2_ + FAN_2_ = 1

FIO_2_ + FICO_2_ + FIN_2_ = 1

Vi: Inspired volume

Va: expired volume

FIO_2_: fractional inspired oxygen

FAO_2_: fractional expired oxygen

FICO_2_: fractional inspired carbon dioxide

FACO_2_: fractional expired carbon dioxide

FIN_2_: fractional inspired nitrogen

FAN_2_: fractional expired nitrogen

Q: Blood flow

CaO_2_: Oxygen content of postcapillary blood

CvO_2_: Oxygen content of mixed venous blood

CaCO_2_: Carbon dioxide content of postcapillary blood

CvCO_2_: carbon dioxide content of mixed venous blood

CaN_2_: nitrogen content of postcapillary blood

CvN_2_: nitrogen content of mixed venous blood

1. Alveolar Dead Space (Alv DS as %)

ideal PACO2 = Partial pressure of carbon dioxide in ideal alveoli (mm Hg)

mixedPACO2 = Mixed expired partial pressure of carbon dioxide prior to mixing with anatomical

space. Derived from expired gas of lung units.

PICO2 = partial pressure of inspired carbon dioxide

1. Expired gas partial pressure

$$Expired gas partial pressure=\left( \sum_{1}^{NC} Vexp x Alv\_Pgas \right)/\sum_{1}^{NC} Vexp$$

Vexp = expired volume of gas from individual lung unit

Alv_Pgas = alveolar partial pressure of gas for individual lung unit

NC = number of compartments

Output

Having characterized the pattern of gas exchange defined by the above parameters the following results are generated:

1. For arterial blood: PaO_2_, O_2_ saturation, O_2_ content, PaCO_2_, CO_2_ content, partial pressure of nitrogen, nitrogen content, pH.
2. For mixed venous blood: mixed venous PO_2_, mixed venous O_2_ saturation, mixed venous O_2_ content, mixed venous PCO_2_, mixed venous CO_2_ content, mixed venous PN_2_, mixed venous nitrogen content, mixed venous pH
3. Values for arterial and mixed venous bicarbonate concentration are calculated. (Equation 4)
4. For ventilation: the total alveolar ventilation (either as at setup or as adjusted to achieve the required PaCO_2_ or pH), mixed alveolar partial pressure of O_2_ and mixed alveolar partial pressure of CO_2_ (Equation 16). These values for O_2_ and CO_2_ represent expired partial pressures before mixing with anatomical dead space.
5. In addition to global data, data for individual lung units are available including values for blood flow, inspired volume, expired volume, O_2_ uptake, CO_2_ excretion, alveolar partial pressures of O_2_, CO_2_ and N_2_, and post capillary partial pressures of O_2_, CO_2_ and nitrogen.
6. R ratio is the ratio of CO_2_ production to O_2_ consumption. An R ratio is calculated for each lung unit. Ideal lung units are defined as those with an R ratio the same as the overall R ratio [1]. Corresponding values of this lung unit represent the ideal VQ ratio, ideal alveolar partial pressure of O_2_ and CO_2_, and ideal lung unit blood O_2_ and CO_2_ content. The ideal lung unit blood content of O_2_ is used in the calculation of venous admixture instead of the conventional approach of relying upon an estimate derived from the alveolar gas equation (Equation 9). The ideal lung unit alveolar partial pressure of CO_2_ is substituted into a version of the Bohr equation to calculate alveolar dead space (Equation 15).
7. For Vi models, the critical VQ ratio represents the VQ ratio for compartments below which negative expired lung volumes occur.

References

DOUGLAS, A. R., JONES, N. L. & REED, J. W. 1988. Calculation of whole blood CO2 content. *J Appl Physiol (1985),* 65**,** 473-7.

KELMAN, G. R. 1967. Digital computer procedure for the conversion of PCO2 into blood CO2 content. *Respir Physiol,* 3**,** 111-5.

MCHARDY, G. J. 1967. The relationship between the differences in pressure and content of carbon dioxide in arterial and venous blood. *Clin Sci,* 32**,** 299-309.

MORGAN, T. J., SCOTT, P. H., LANGLEY, A. N., BARRETT, R. D. C. & ANSTEY, C. M. 2023. Single-FiO(2) lung modelling with machine learning: a computer simulation incorporating volumetric capnography. *J Clin Monit Comput*.

SIGGAARD-ANDERSEN, O. 1977. The Van Slyke equation. *Scand J Clin Lab Invest Suppl,* 37**,** 15-20.

SIGGAARD-ANDERSEN, O., SIGGAARD-ANDERSEN, M. & FOGH-ANDERSEN, N. 1993. The TANH-equation modified for the hemoglobin, oxygen, and carbon monoxide equilibrium. *Scand J Clin Lab Invest Suppl,* 214**,** 113-9.

WAGNER, P. D. 2008. The multiple inert gas elimination technique (MIGET). *Intensive Care Med,* 34**,** 994-1001.

WEST, J. B. 1969. Ventilation-perfusion inequality and overall gas exchange in computer models of the lung. *Respir Physiol,* 7**,** 88-110.

WEST, J. B., WAGNER, PD 1977. Pulmonary gas exchange. *In:* WEST, J. B. (ed.) *Bioengineering Aspects of the Lung.* New York: Marcel Dekker.
